# Socio-economic status, visual impairment and the mediating role of lifestyles in developed rural areas of China

**DOI:** 10.1371/journal.pone.0215329

**Published:** 2019-04-11

**Authors:** Xiaochang Yan, Lu Chen, Hua Yan

**Affiliations:** 1 School of Economics, Peking University, Beijing, China; 2 School of Finance, Nankai University, Tianjin, China; 3 Department of Ophthalmology, Tianjin Medical University General Hospital, Tianjin, China; Wenzhou Medical University, CHINA

## Abstract

**Purpose:**

To examine the impacts of socio-economic status (SES) on visual impairment (VI), and the mediating role of lifestyles in developed rural areas of China.

**Methods:**

A cross-sectional study was conducted among people living in rural districts of Tianjin, a developed municipality in China. An interviewer-administered survey along with free eye screenings was conducted with participants. The questionnaire included questions about demographic characteristics, SES, medical histories and lifestyles. Presenting visual acuity (PVA) and main causes of VI were identified by ophthalmologists. χ2 test was undertaken to determine whether significant differences (p<0.05) exist between VI and demographic, SES, medical history and lifestyle factors. A stepwise regression method was conducted to investigate whether lifestyles play mediating roles between SES and VI. Multivariable logistic and ordinal logistic regression were used contingent on different types of dependent variables in each regression, and adjusted odds ratio (OR) values were estimated.

**Results:**

Of the 12,233 participants, 6,233 were male (50.59%); the mean age was 34.61 years; 310 (2.54%) had VI. Hypertension, diabetes and cardiopathy were main medical histories, with 1,640 had hypertension (13.41%), 854 had diabetes (6.98%) and 483 had cardiopathy (3.95%). About SES factors, higher education level (Adjusted OR, 0.84; 95% CI, 0.75–0.95) and higher income level (Adjusted OR, 0.54; 95% CI, 0.39–0.76), were significantly associated with VI in a gradient across severity of VI. Lifestyles including smoking (Adjusted OR, 1.55; 95% CI, 1.31–1.83) and drinking (Adjusted OR, 1.36; 95% CI, 1.06–1.74) played mediating roles between SES and VI when considering the full sample. Besides smoking and drinking, reading every week (Adjusted OR, 2.07; 95% CI, 1.53–2.82) and exercising more than 2h every day (Adjusted OR, 0.39; 95% CI, 0.15–1.00) also played mediating roles between SES and VI when considering the subsample (age≥16).

**Conclusion:**

This study revealed the crucial impacts of SES factors on VI, and the mediating role played by several lifestyles. Targeted public health interventions for reducing VI should thus be proposed in developed rural areas of China.

## Introduction

Visual impairment (VI) constitutes a major public health problem. It has caused significant suffering, disability, poor mental health, increased cognitive deterioration, a deterioration in quality of life, loss of productivity and enormous economic consequences for millions of people around the world [1–4]. Moreover, the majority of people with VI live in developing countries, and visual problems are usually more prevalent and serious in rural areas of a country [5]. As a disability, VI greatly exacerbates the issue of productivity loss, instigating considerable financial pressure to such countries and individuals alike [6].

More promising is the fact that more than two thirds of VI could be avoided through either prevention or treatment [7]. In order to increase the effectiveness of the prevention and treatment of VI, several principal risk factors of VI have been identified, including ageing, gender and certain medical histories [8–10]. The prevalence of VI is unequally distributed throughout the world and has been found to be strongly associated with socio-economic status (SES) [11–14]. The strong correlation between SES and VI is particularly true in many rapidly developing countries in Asia, such as China that are demonstrating growing gaps in SES, which is measured in terms of income and education [15]. In addition, social epidemiology studies have noted that lifestyle has a significant impact on people’s health [16]. Lifestyle has been identified as one of the intermediate mechanisms that links SES with health, that is, differences in SES lead to inequalities in health through lifestyle [17]. As an important aspect of physical health, visual health is also affected by people’s lifestyles. However, relatively little research has examined the mediating role of lifestyle factors between SES and VI.

As a rapidly growing country, China is facing serious imbalances in its economic and social development. In recent decades, the prevalence of VI has increased in China, and is unequally distributed among groups with different SES. By 2017, the number of people with VI in China reached 13 million, of whom about 4 million are blind due to cataracts. Cataracts were found to represent one of the leading causes of blindness among visually impaired people in China. In 2016, the “13^th^ Five-Year National Eye Health Plan (2016–2020)” formulated by the National Health and Family Planning Committee of China emphasised the importance of visual health [18]. To improve people’s visual health, the Communist Party of China (CPC) Central Committee and the State Council formulated the Healthy China Strategy, and repeatedly and fastidiously mentioned its contents regarding “focusing on improving the health of whole people and popularizing healthy lifestyles” in the “Healthy China 2030” planning outline [19]. Of course, more attention should be paid to the impact of SES on visual health from the perspective of improving people’s lifestyles.

The aim of the current study was to examine the effects of SES on VI using samples from developed rural areas of China, and to further analyse the mediating effects of several lifestyle factors on this relationship. Several public health interventions for reducing VI were proposed in the end.

## Materials and methods

### Study population, sample and data collection

This cross-sectional study was conducted from November 2015 to March 2017 on a Chinese population living in Tianjin, a developed municipality in Northern China. The rural districts included Wuqing, Binhai New District, Jizhou and Jinghai, which are located in the westernmost, easternmost, northernmost and southernmost parts of Tianjin, respectively.

All the participants of this survey were recruited in October 2015. We conducted multistage cluster sampling with persons of all age groups in the selected districts. We divided all ages into three groups (ranges: 1–6, 7–15, ≥16 years). The division of age groups was based on Chinese criteria, which define people aged 0 to 6 as preschoolers, people aged 7 to 15 as school-aged children [20], and people aged 16 and above as adults. There was no evidence within the same study setting in which we should consider estimating the minimum sample size. According to data released by the China Disabled Persons Federation (CDPF), we estimated that the expected prevalence of VI in developed rural areas of Tianjin is 4%. Based on the formula of simple random sampling, we calculated the sample size for each age group, with a 95% confidence interval (CI), an allowable error bound of 20% and a 10% non-response rate. A sample size of 5,122 individuals was calculated for each age group, and the required sample size of all age groups was ultimately estimated at 15,366. When choosing clusters, 10 clusters were randomly selected for each district, with a probability proportionate to size and the systematic random sampling method. Sampling within each cluster considered reasonable proportions of age and sex, and continued until at least 384 individuals had entered the study, resulting in a sample size of approximately 3,842 for each district.

SES and demographic characteristics were measured by a well-designed questionnaire (See S1 and S2 Appendices). The questionnaire included 11 questions covering demographic data, SES data, medical history and lifestyle information. The order of questions was such that individuals were asked about age, sex and address first, and subsequently marital status, education level, occupation and income level. They were next asked about their relevant ocular and medical histories, frequency of reading and doing physical exercise, and whether they partake in living habits such as smoking, drinking and staying up late. In order to assess its applicability, the questionnaire had been tested with a pilot sample of 20 people prior to data collection. Health workers asked the participants questions and controlled the validity of answers. To improve the accuracy and effectiveness of the data, the health workers studied relevant protocols and were trained about interview methods before data collection.

In order to collect eye examination data, senior ophthalmologists visited all participants and identified visual acuity and lens status through a tumbling E chart and a direct ophthalmoscope. For children under 5 years old, HOTV cards were used and appropriate visual acuity equivalents were recorded for analysis. Regardless, presenting visual acuity (PVA) was recorded for each participant. Subsequently, for the participants with PVA of less than 6/18 in either eye, an ophthalmologist performed slit-lamp biomicroscopy and direct ophthalmoscopy through a dilated pupil. The main causes of VI were identified by the ophthalmologists and recorded on the customised form listed on the back of the questionnaires. We had access to information that could identify individual participants after data collection.

### Definitions

In this study, the definition of VI was based on that of the World Health Organization (WHO) and was reported based on best corrected PVA in the better eye. VI included blindness and low vision. Blindness was defined as PVA of less than 3/60 in the better eye. Moreover, blindness could be further divided into two categories: profound visual impairment (PVI) (12/600≤PVA<3/60) and near total blindness (NTB) (PVA<12/600). Low vision was defined as PVA between 3/60 and 6/18. Low vision could also be further divided into two categories: severe visual impairment (SVI) (3/60≤PVA<6/60) and moderate visual impairment (MVI) (6/60≤PVA<6/18).

### Variables

Table 1 shows the definitions and values of all the variables. The outcome variable of interest was VI. Based on the definitions of VI mentioned above, we scored VI from 0 to 4 according to best corrected PVA in the better eye. Specifically, participants with PVA<12/600 were defined as NTB, and we scored their VI as 4; participants with 12/600≤PVA<3/60 were defined as PVI, and we scored their VI as 3; participants with 3/60≤PVA<6/60 were defined as SVI, and we scored their VI as 2; participants with 6/60≤PVA<6/18 were defined as MVI, and we scored their VI as 1; all other participants were defined as NVI, and we scored their VI as 0.

**Table 1:** Definitions and values of variables.

Variables	Definition	Value
**VI**	VI was scored from 0 to 4 according to best corrected PVA in the better eye.	0 = NVI (PVA≥6/18), 1 = MVI (6/60≤PVA<6/18), 2 = SVI (3/60≤PVA<6/60), 3 = PVI (12/600≤PVA<3/60) and 4 = NTB (PVA<12/600)
**Age**	Age when filling out the questionnaire	Range from 1 to 94 years
**Sex**	Male or female	0 = Male, 1 = Female
**District**	The district of the participant's address	0 = Wuqing, 1 = Binhai New District, 2 = Jixian, 3 = Jinghai
**Education Level**	Average years of studies according to education level	0 = No studies, 4 = Primary, 8 = Junior, 11 = Senior, 15 = University and above
**Income Level**	Per capita household income per month reported in five categories	0 = No income, 1 = Up to 1,000 yuan, 2 = 1,000–2,000 yuan, 3 = 2,000–5,000 yuan, 4 = More than 5,000 yuan
**Medical Histories**		
Hypertension	Whether the participant had ever had hypertension	0 = No, 1 = Yes
Diabetes	Whether the participant had ever had diabetes	0 = No, 1 = Yes
Cardiopathy	Whether the participant had ever had cardiopathy	0 = No, 1 = Yes
CT	Whether the participant had ever had CT	0 = No, 1 = Yes
Tumour	Whether the participant had ever had tumour	0 = No, 1 = Yes
**Lifestyles**		
Smoking	Whether the participant smoke	0 = No, 1 = Yes
Drinking	Whether the participant drink alcohol	0 = No, 1 = Yes
Staying up Late	Whether the participant often stay up late	0 = No, 1 = Yes
Reading	Frequency of reading	0 = Never, 1 = Every week, 2 = Less than 1h/day, 3 = 1-2h/day, 4 = More than 2h/day
Exercise	Frequency of exercising	1 = Less than 1h/day, 2 = 1-2h/day, 3 = More than 2h/day

CT, Cerebral trauma; MVI, Moderate visual impairment; NTB, Near total blindness; NVI, No visual impairment; PVA, Presenting visual acuity; PVI, Profound visual impairment; SVI, Severe visual impairment; VI, Visual impairment.

In terms of independent variables, this study mainly included three categories. The first category comprised demographic factors, including age (ranges: 1–6, 7–15, ≥16 years) and sex (male, female). The second category comprised SES factors. Given that education and income are the most commonly studied determinants of SES in all literatures [21,22], we used education level (average years of studies according to different education levels, including no studies, primary, junior, senior, university and above) and income level (per capita household income per month: no income, up to 1,000 yuan, between 1,000 and 2,000 yuan, between 2,000 and 5,000 yuan, more than 5,000 yuan) as the proxy variables of SES. The income groups were roughly divided by reference to the data on per capita monthly disposable income from 2015 to 2017. Since our study focused on the developed rural area in China and was conducted in Tianjin, we mainly referred to the data on per capita monthly disposable income of rural households in Tianjin (1,540 yuan in 2015, 1,673 yuan in 2016 and 1,813 yuan in 2017) and national average (990 yuan in 2015, 1,073 yuan in 2016 and 1,167 yuan in 2017), with all the data between 1,000 yuan and 2,000 yuan [23]. To make the division of income groups in line with the situation in developed rural area in China, and to ensure a reasonable distribution of samples across all possible income groups, we also referred to the data of urban households, data of urban and rural households, and data of other provinces in China, which helped us set a third threshold of 5,000 (See S2 Table). The third category of independent variables is medical history, including hypertension, diabetes, cardiopathy, cerebral trauma (CT) and tumour.

To explore the mediating effects of lifestyles, we also considered independent variables of lifestyle, including smoking, drinking, staying up late, reading (never, every week, less than 1h every day, between 1h and 2h every day, more than 2h every day) and exercising (less than 1h every day, between 1h and 2h every day, more than 2h every day).

### Statistical analysis

Descriptive analysis of the distribution of VI was based on all participants, and was conducted through the calculation of frequencies and ratios. To determine whether statistically significant differences (p<0.05) exist between VI and demographic, SES, medical history and lifestyle factors, the χ^2^ test was then undertaken.

In order to investigate whether lifestyles play mediating roles between SES and VI, a stepwise regression method was conducted. Multivariable logistic and ordinal logistic regression were used contingent on different types of dependent variables in each regression. According to the stepwise regression method proposed by Baron and Kenny, we had to measure the effect of SES on VI in the first step [24]. We regressed SES variables on VI, controlling for demographic and medical history variables. In the second step, to measure the effect of SES on each lifestyle, we regressed SES variables on each lifestyle variable, controlling for demographic and medical history variables. Among the 5 regressions in this step, only those with statistically significant SES variables meant that the corresponding lifestyle variables might play mediating roles and were worthy of being researched in depth. Therefore, in the third step, we regressed SES variables and the selected lifestyle variables on VI, controlling for demographic and medical history variables. Through analysing the significance level of lifestyle variables, we were ultimately able to ascertain which lifestyles play mediating roles between SES and VI. Robust SEs were used to account for the clustering of individuals within each district. In addition, given that preschool-aged children may receive little education, and most school-aged children may continue to receive education and have no income, we undertook an additional subgroup analysis restricted to those participants who were 16 years old and above. Statistical analyses were performed using Stata version 14.0 (Stata Corp, College Station, Texas, USA).

### Ethical issues

The study adhered to the tenets of the Declaration of Helsinki and was approved by the Ethics Committee of Tianjin Medical University of Medical Science. Prior to the enrollment, the goals, importance and procedures of the study were conveyed to all participants through designed forms, and written informed consent was obtained from everybody. The privacy and confidentiality of all participants were secured.

## Results

Table 2 displays the number of participants, distribution of demographic, SES, medical history and lifestyle indicators of all participants, and people with VI or no visual impairment (NVI). Of a total of 15,368 participants in the four districts, 12,233 were included for final analysis having excluded those with unknown outcomes and incomplete questionnaires (response rate: 79.60%). The age of participants averaged 34.61 years and ranged from 1 to 94 years. The majority of participants were male (50.95%), while the majority of people with VI were female (63.23%). Most participants had a low education level and income level, of whom 4,267 participants (34.88%) were at primary education level and 7,126 participants (58.25%) had monthly incomes below 1,000 yuan. Among all medical histories, hypertension, diabetes and cardiopathy were mentioned most frequently. Specifically, 1,640 participants (13.41%) had hypertension, 854 participants (6.98%) had diabetes, and 483 participants (3.95%) had cardiopathy. Among all participants, a total of 11,923 (97.47%) had NVI, 293 (2.40%) had low vision, and 17 (0.14%) were blind.

**Table 2:** Distribution of demographic, socio-economic status, medical history and lifestyle variables among all participants and people with or without visual impairment.

Variables	Total^†^	NVI^†^	VI^†^
	n = 12,233(%)	n = 11,923(%)	n = 310(%)
**Age (years)**			
1–6	943(7.71)	943(7.91)	0(0)
7–15	3,449(28.19)	3,443(28.88)	6(1.94)
≥16	7,841(64.10)	7,537(63.21)	304(98.06)
**Sex**			
Male	6,233(50.95)	6,119(51.32)	114(36.77)
Female	6,000(49.05)	5,804(48.68)	196(63.23)
**District**			
Wuqing	2,466(20.16)	2,459(20.62)	7(2.26)
Binhai	3,894(31.83)	3,729(31.28)	165(53.23)
Jixian	2,852(23.31)	2,769(23.22)	83(26.77)
Jinghai	3,021(24.70)	2,966(24.88)	55(17.74)
**Education Level**			
No Studies	1,596(13.05)	1,517(12.72)	79(25.48)
Primary	4,267(34.88)	4,117(34.53)	150(48.39)
Junior	2,576(21.06)	2,537(21.28)	39(12.58)
Senior	2,398(19.60)	2,376(19.93)	22(7.10)
University & Above	1,396(11.41)	1,376(11.54)	20(6.45)
**Income Level**			
No Income	5,315(43.45)	5,227(43.84)	88(28.39)
Up to 1,000 yuan	1,811(14.80)	1,663(13.95)	148(47.74)
1,000–2,000 yuan	1,491(12.19)	1,466(12.30)	25(8.06)
2,000–5,000 yuan	2,390(19.54)	2,356(19.76)	34(10.97)
More than 5,000 yuan	1,226(10.02)	1,211(10.16)	15(4.84)
**Medical History**			
Hypertension	1,640(13.41)	1,506(12.63)	134(43.23)
Diabetes	854(6.98)	779(6.53)	75(24.19)
Cardiopathy	483(3.95)	429(3.60)	54(17.42)
CT	34(0.28)	29(0.24)	5(1.61)
Tumour	106(0.87)	92(0.77)	14(4.52)
**Lifestyles**			
Smoking	1,154(9.43)	1,099(9.22)	55(17.74)
Drinking	873(7.14)	828(6.94)	45(14.52)
Staying up Late	2,085(17.04)	2,057(17.25)	28(9.03)
Reading			
Never	1,627(13.30)	1,600(13.42)	27(8.71)
Every Week	3,511(28.70)	3,377(28.32)	134(43.23)
<1h/day	2,553(20.87)	2,457(20.61)	96(30.97)
1-2h/day	4,490(36.70)	4,438(37.22)	52(16.77)
>2h/day	52(0.43)	51(0.43)	1(0.32)
Exercise			
<1h/day	4,013(32.80)	3,782(31.72)	231(74.52)
1-2h/day	4,098(33.50)	4,053(33.99)	45(14.52)
>2h/day	4,122(33.70)	4,088(34.29)	34(10.97)

CT, Cerebral trauma; NVI, No visual impairment; VI, Visual impairment.

^†^Counts and percentages are presented unless otherwise stated.

### Severity of visual impairment according to demographic factors, socio-economic status, medical history and lifestyle

To more specifically analyse the relationship between SES and severity of VI, we further classified the 310 participants with VI into four levels. Of the participants with low vision, 261 (89.08%) had MVI and 32 (10.92%) had SVI. Of the participants who were blind, 9 (52.94%) had PVI and 8 (47.06%) had NTB.

Table 3 shows the distribution of demographic, SES, medical history and lifestyle variables among the participants in four VI levels. The distribution of age, sex, education level, income level, medical histories (hypertension, diabetes, cardiopathy, CT and tumour) and lifestyles (smoking, drinking, staying up late, reading and exercise) differed considerably among the four VI levels (all P <0.01), which means that all of the above factors were significantly associated with VI.

**Table 3:** Distribution of demographic, socio-economic status, medical history and lifestyle variables stratified by the severity of visual impairment.

Variables	Total VI^†^ n = 310(%)	MVI^†^ n = 261(%)	SVI^†^ n = 32(%)	PVI^†^ n = 9(%)	NTB^†^ n = 8(%)	χ2	P Value^‡^
**Age(years)**						704.75	0.000
1–6	0(0)	0(0)	0(0)	0(0)	0(0)		
7–15	6(1.94)	6(2.30)	0(0)	0(0)	0(0)		
≥16	304(98.06)	255(97.70)	32(100.0)	9(100.0)	8(100.0)		
**Sex**						27.36	0.000
Male	114(36.77)	98(37.55)	10(31.25)	2(22.22)	4(50.00)		
Female	196(63.23)	163(62.45)	22(68.75)	7(77.78)	4(50.00)		
**Education Level**						109.35	0.000
No Studies	79(25.48)	69(26.44)	8(25.00)	0(0)	2(25.00)		
Primary	150(48.39)	119(45.59)	19(59.38)	6(66.67)	6(75.00)		
Junior	39(12.58)	35(13.41)	2(6.25)	2(22.22)	0(0)		
Senior	22(7.10)	19(7.28)	2(6.25)	1(11.11)	0(0)		
University & Above	20(6.45)	19(7.28)	1(3.13)	0(0)	0(0)		
**Income Level**						286.04	0.000
No Income	88(28.39)	77(29.50)	8(25.00)	1(11.11)	2(25.00)		
Up to 1,000 yuan	148(47.74)	118(45.21)	18(56.25)	6(66.67)	6(75.00)		
1,000–2,000 yuan	25(8.06)	22(8.43)	2(6.25)	1(11.11)	0(0)		
2,000–5,000 yuan	34(10.97)	30(11.49)	3(9.38)	1(11.11)	0(0)		
More than 5,000 yuan	15(4.84)	14(5.36)	1(3.13)	0(0)	0(0)		
**Medical History**							
Hypertension	134(43.23)	109(41.76)	16(50.00)	5(55.56)	4(50.00)	246.86	0.000
Diabetes	75(24.19)	64(24.52)	7(21.88)	3(33.33)	1(12.50)	148.26	0.000
Cardiopathy	54(17.42)	45(17.24)	6(18.75)	1(11.11)	2(25.00)	154.52	0.000
CT	5(1.61)	3(1.15)	1(3.13)	0(0)	1(12.50)	60.17	0.000
Tumour	14(4.52)	14(5.36)	0(0)	0(0)	0(0)	63.14	0.000
**Lifestyles**							
Smoking	55(17.74)	49(18.77)	3(9.38)	2(22.22)	1(12.50)	29.11	0.000
Drinking	45(14.52)	39(14.94)	5(15.63)	1(11.11)	0(0)	28.97	0.000
Staying up Late	28(9.03)	22(8.43)	3(9.38)	2(22.22)	1(12.50)	15.68	0.003
Reading						117.39	0.000
Never	27(8.71)	20(7.66)	6(18.75)	0(0)	1(12.50)		
Every Week	134(43.23)	129(49.43)	4(12.50)	0(0)	1(12.50)		
<1h/day	96(30.97)	72(27.59)	15(46.88)	6(66.67)	3(37.50)		
1-2h/day	52(16.77)	39(14.94)	7(21.88)	3(33.33)	3(37.50)		
>2h/day	1(0.32)	1(0.38)	0(0)	0(0)	0(0)		
Exercise						259.55	0.000
<1h/day	231(74.52)	198(75.86)	19(59.38)	8(88.89)	6(75.00)		
1-2h/day	45(14.52)	40(15.33)	3(9.38)	1(11.11)	1(12.50)		
>2h/day	34(10.97)	23(8.81)	10(31.25)	0(0)	1(12.50)		

CT, Cerebral trauma; MVI, Moderate visual impairment; NTB, Near total blindness; PVI, Profound visual impairment; SVI, Severe visual impairment; VI, Visual impairment.

^†^Counts and percentages are presented unless otherwise stated.

^‡^P Value was based on Pearson χ2 test.

### Socio-economic status and visual impairment

Even if we can make a preliminary judgment regarding the impact of SES and lifestyles on VI from the results of Table 3, we are currently unable to identify the specific effects of SES on VI, and whether lifestyle factors play mediating roles between SES and VI. Therefore, we undertook a stepwise regression method for further analysis. In the first step, to measure the effect of SES on VI, we regressed SES variables on VI using ordinal logistic regression, with demographic and medical history variables controlled. Table 4 presents the effects of demographic factors, SES and medical history on VI. Specifically, the risk of a female having VI was 1.33 times that of a male at a 1% significance level. For each additional year of age, the risk of VI increasing by one level significantly increased by 0.09 times (P<0.01). However, for each additional level of education and income, the risk of VI increasing by one level significantly decreased by 0.16 times and 0.46 times, respectively (P<0.01). As regards medical histories, the risks of VI in participants with hypertension, diabetes, CT and tumour were respectively 1.20, 1.20, 1.36 and 2.28 times those of the participants without the above medical histories (P<0.1).

**Table 4:** Effects of demographic factors, socio-economic status and medical history on visual impairment.

Variables	All Participants		Age≥16	
	Adjusted OR^^^	95% CI	Adjusted OR^^^	95% CI
**Sex**^**†**^				
Male	1 [Reference]		1 [Reference]	
Female	1.33***	1.08–1.63	1.31**	1.04–1.65
**Age (years)**	1.09***	1.07–1.11	1.09***	1.07–1.11
**Education Level**^**†**^				
No Studies	1 [Reference]		1 [Reference]	
Increasing Education Level	0.84***	0.75–0.95	0.86***	0.78–0.96
**Income Level**^**†**^				
No Income	1 [Reference]		1 [Reference]	
Increasing Income Level	0.54***	0.39–0.76	0.59***	0.43–0.80
**Medical Histories**				
Hypertension	1.20***	1.15–1.25	1.20***	1.15–1.24
Diabetes	1.20**	1.03–1.40	1.20**	1.03–1.40
Cardiopathy	0.98	0.50–1.90	0.98	0.50–1.91
CT	1.36*	0.97–1.90	1.36*	0.96–1.93
Tumour	2.28***	1.89–2.75	2.28***	1.89–2.74

CI, Confidence interval; CT, Cerebral trauma; OR, Odds ratio.

^**^**^Ordinal logistic regression adjusted for age, sex, education level, income level and medical histories.

^†^Reference categories of variables (Sex, Education Level and Income Level) are Male, No Studies and No Income, respectively.

* P<0.1

** P<0.05

*** P<0.01.

To ensure the robustness of the above results, we undertook an additional subgroup analysis restricted to participants who were 16 years old and above. The results of the subgroup analysis were basically consistent with the conclusions of the full sample analysis. Therefore, we concluded that increased VI was significantly associated with decreased education level and decreased income level.

### Socio-economic status and lifestyle

In the second step, to measure the effect of SES on different lifestyles, we regressed SES variables on each lifestyle variable, using either multivariable logistic or ordinal logistic regression contingent on the type of dependent variable in each regression, with demographic and medical history variables controlled. Table 5 shows the effects of SES on lifestyle. Full sample analysis indicated that the risk of staying up late significantly decreased with increasing level of education (odds ratio [OR]<1, P<0.01), and the risk of smoking and drinking significantly rose with increasing level of income (OR>1, P<0.01). Given that both education level and income level were defined as SES factors, we concluded that SES had significant impacts on smoking, drinking and staying up late.

**Table 5:** Effects of socio-economic status on lifestyle.

	**Adjusted OR (95% IC)**^**^**^ **- All Participants**				
**Variables**	**Smoking**^**a**^	**Drinking**^**b**^	**Staying up Late**^**c**^	**Reading**^**d**^	**Exercise**^**e**^
**Education Level**^**†**^					
No Studies	1 [Reference]	1 [Reference]	1 [Reference]	1 [Reference]	1 [Reference]
Increasing Education Level	0.99 (0.96–1.02)	0.99 (0.95–1.03)	0.81*** (0.79–0.84)	0.94 (0.76–1.15)	0.93 (0.78–1.11)
**Income Level**^**†**^					
No Income	1 [Reference]	1 [Reference]	1 [Reference]	1 [Reference]	1 [Reference]
Increasing Income Level	1.60*** (1.46–1.77)	1.52*** (1.30–1.78)	0.97 (0.70–1.34)	0.73 (0.44–1.20)	0.78 (0.47–1.30)
	**Adjusted OR (95% IC)**^**^**^ **- Age≥16**				
**Variables**	**Smoking**	**Drinking**	**Staying up Late**	**Reading**	**Exercise**
**Education Level**					
No Studies	1 [Reference]	1 [Reference]	1 [Reference]	1 [Reference]	1 [Reference]
Increasing Education Level	1.03 (0.96–1.10)	1.02 (0.99–1.05)	1.03 (0.99–1.07)	0.95 (0.90–1.01)	1.03 (0.95–1.12)
**Income Level**					
No Income	1 [Reference]	1 [Reference]	1 [Reference]	1 [Reference]	1 [Reference]
Increasing Income Level	1.29*** (1.19–1.40)	1.30*** (1.09–1.54)	1.05 (0.89–1.24)	0.85*** (0.78–0.94)	1.13** (1.00–1.26)

CI, Confidence interval; OR, Odds ratio.

^a^Logistic regression with reference category (not smoking).

^b^Logistic regression with reference category (not drinking).

^c^Logistic regression with reference category (not staying up late).

^d^Ordinal logistic regression—5 categories of frequency at which participants read.

^e^Ordinal logistic regression—3 categories of frequency at which participants exercise.

^^^All estimates were adjusted for age, sex, medical histories, education level and income level.

^†^Reference categories of variables (Education Level and Income Level) are No Studies and No Income, respectively.

** P<0.05

*** P<0.01.

However, the results of the subgroup analysis were not exactly the same. The risk of smoking, drinking and exercising significantly rose with increasing level of income (OR>1, P<0.05), while the risk of reading was significantly inversely associated with increasing level of income (OR<1, P<0.01). Moreover, education level was demonstrated to have no significant impact on any lifestyle variables. Therefore, among participants aged≥16, we could conclude that SES had significant impacts on smoking, drinking, reading and exercising.

### Mediating effect of lifestyles between socio-economic status and visual impairment

From the previous step, only the lifestyles that demonstrated a significant association with SES may play mediating roles between SES and VI. Therefore, in the third step, to measure the mediating effect of lifestyles, we regressed SES variables and the selected lifestyle variables on VI using ordinal logistic regression, with demographic and medical history variables controlled. Table 6 presents the effects of demographic factors, SES, medical history and lifestyles on VI for both the full sample and the subsample. Furthermore, the results of step 1 were also shown for convenient comparison. The results of demographic factors, SES and medical histories in step 3 were basically consistent with those in step 1. In particular, increased VI showed significant correlations with decreased education level and decreased income level (OR<1, P<0.01).

**Table 6:** Mediating role of lifestyles between socio-economic status and visual impairment.

Variables	VI-Adjusted OR (95% CI)^^^			
	All Participants		Age≥16	
	Step 1	Step 3	Step 1	Step 3
**Sex**^**†**^				
Male	1 [Reference]	1 [Reference]	1 [Reference]	1 [Reference]
Female	1.33*** (1.08 to 1.63)	1.36*** (1.11–1.66)	1.31** (1.04 to 1.65)	1.36** (1.07–1.73)
**Age (years)**	1.09*** (1.07 to 1.11)	1.09*** (1.07–1.11)	1.09*** (1.07 to 1.11)	1.09*** (1.07–1.11)
**Education Level**^**†**^				
No Studies	1 [Reference]	1 [Reference]	1 [Reference]	1 [Reference]
Increasing Education Level	0.84*** (0.75 to 0.95)	0.83*** (0.75–0.94)	0.86*** (0.78 to 0.96)	0.85*** (0.77–0.94)
**Income Level**^**†**^				
No Income	1 [Reference]	1 [Reference]	1 [Reference]	1 [Reference]
Increasing Income Level	0.54*** (0.39 to 0.76)	0.53*** (0.38–0.74)	0.59*** (0.43 to 0.80)	0.56*** (0.40–0.79)
**Medical Histories**				
Hypertension	1.20*** (1.15 to 1.25)	1.19*** (1.15–1.23)	1.20*** (1.15 to 1.24)	1.15** (1.02–1.30)
Diabetes	1.20** (1.03 to 1.40)	1.21*** (1.05–1.40)	1.20** (1.03 to 1.40)	1.20*** (1.11–1.29)
Cardiopathy	0.98 (0.50 to 1.89)	0.97 (0.49–1.92)	0.98 (0.50 to 1.91)	1.00 (0.51–1.95)
CT	1.36* (0.97 to 1.90)	1.49** (1.06–2.09)	1.36* (0.96 to 1.93)	1.89** (1.04–3.41)
Tumour	2.28*** (1.89 to 2.75)	2.34*** (1.92–2.86)	2.28*** (1.89 to 2.74)	2.27*** (1.79–2.90)
**Lifestyles**				
Smoking		1.55*** (1.31–1.83)		1.27* (0.99–1.63)
Drinking		1.36** (1.06–1.74)		1.39** (1.08–1.80)
Staying up Late		1.17 (0.84–1.62)		
Reading^**†**^				
Never				1 [Reference]
Every Week				2.07*** (1.53–2.82)
<1h/day				1.05
				(0.70–1.58)
1-2h/day				1.09
				(0.37–3.19)
>2h/day				0.77
				(0.18–3.36)
Exercise^**†**^				
<1h/day				1 [Reference]
1-2h/day				0.79
				(0.41–1.54)
>2h/day				0.39* (0.15–1.00)

CI, Confidence interval; CT, Cerebral trauma; OR, Odds ratio.

^^^Ordinal logistic regression adjusted for age, sex, education level, income level and medical histories.

^†^Reference categories of variables (Sex, Education Level, Income Level, Reading and Exercise) are Male, No Studies, No Income, Never Reading and Exercise Less than 1h/day, respectively.

* P<0.1

** P<0.05

*** P<0.01.

When considering the full sample, the risk of VI in participants who smoke and drink were respectively 1.55 and 1.36 times that of the participants without these lifestyles. However, the subgroup analysis showed that in addition to smoking and drinking, reading and exercising also had significant effects on VI. Firstly, the risk of VI in the participants who read every week was 2.07 times that of the participants who had no reading (OR>1, P<0.01). Although weekly reading was shown to increase the risk of VI, higher frequencies of reading were shown to have no significant effect on VI. This result might be due to the characteristics of the study sample’s lifestyle. As shown in Table 3, only 54 participants read for 1–2 hours every day (16.77%) and 1 participant read for more than 2 hours every day (0.32%). Since the sample with higher frequencies of reading was too small, it was very likely to make the results statistically insignificant. Secondly, the risk of VI in the participants who exercised more than two hours every day was 0.39 times that of the participants who exercised less than one hour every day (OR<1, P<0.1), which means that exercising with higher frequency could significantly reduce the risk of VI. Therefore, we were able to broadly conclude that smoking, drinking, reading and exercising play mediating roles between SES and VI.

## Discussion

In this large sample of participants in developed rural areas of China, we investigated the association of SES and VI, as well as the mediating role of lifestyles. We found that VI is associated with several key demographic (including age and sex), SES (including educational level and income level) and medical history factors, with a trend across the severity of VI. Moreover, several lifestyles were demonstrated to have mediating effects between SES and VI. In addition, all of the above results were generally consistent with the results of subgroup analysis (participants excluding preschoolers and school-aged children), without substantial differences.

Of the 12,233 participants analysed, 310 (0.25%) had VI, which may be lower than the prevalence rate in some other studies [25,26]. Importantly, the prevalence of VI is not officially reported and precisely known around the world, and relevant research remains insufficient [11]. Our rate of people with VI is relatively low for the following possible reasons. First, few studies have targeted all age groups, and most previous surveys have used older people as the sample [27]. Given that older people are more likely to have eye problems, the prevalence of VI in such studies is higher than the rate in this study. Second, our sample is not a random subsample of the population in China, hence we cannot interpret frequency and distribution as prevalence. Thus, the frequency of VI may be lower than expected owing to the non-random selection of participants for geographical convenience [28–33]. Third, in our study, VI was strictly defined in accordance with the WHO. However, some studies have used different definitions of VI, restricting the comparability of results [11]. Nevertheless, given that convenience sampling was used, people with VI could be more likely to participate in our study, reducing our underestimation of VI to some extent.

Our study found that older people had a higher risk of VI. As previous studies have already proved, older people have more eye problems due to their overall poorer physical function [34]. Moreover, older people may be more likely to relate their vision capacity to their age and believe that no effective treatment exists, preventing them from receiving treatment and thus increasing their risk of VI, as well as the severity of VI [35].

Females’ higher risk of having VI in our study has also been reported in prior studies [36–38]. Moreover, some studies focusing on children in China and Turkey have also found the prevalence of VI to be higher in girls [39,40]. Pi et al. have argued that females are usually at greater risk of having VI owing to inequitable distribution among the population, females’ lower incomes and social status, as well as some other factors [20].

Our study found that people with hypertension, diabetes, CT and tumour were at greater risk of having VI, as has been shown in other studies [41,42]. The reason is that diseases such as hypertension, diabetes, CT and tumour are usually associated with poor glucose management, lipids in the blood and poor overall physical condition, which have some adverse consequences in terms of eye diseases. Thus, for people with diabetes or certain other chronic diseases, the clinical guidelines recommend an examination including observation of the back of eyes every 1 to 2 years even for those without known eye problems [43].

As two main proxy variables of SES, education level and income level were found to have significant impacts on VI. The majority of previous studies have suggested that people with lower levels of education are more prone to VI [13,44–47], which is consistent with the result of this study. Possible reasons for this result include the following. Education affects people’s visual health through a variety of different mechanisms, such as changing people’s lifestyle, improving people’s ability to solve problems and changing people’s values. Besides, education can promote people’s mental maturity and cultivate their ability to make money [48,49]. Some studies also found that, compared with people with lower education level, people with higher education level generally have more control over their lives and health even under intense work stress [50]. All the reasons mentioned above make us believe that people with a higher education level are usually more knowledgeable about eye care, and tend to pay more attention to avoid activities which are harmful to visual health. In addition, our study found that people with higher incomes had a lower risk of having VI, in line with previous studies [14,51–53]. Low-income populations tend to have less access to eye care services. Even if the availability and affordability of eye care services increase, people with poor economic status continue to utilise these services improperly and insufficiently [52].

Our study has also demonstrated that lifestyles including smoking, drinking, reading and exercising play mediating roles between SES and VI. Previous studies have found that SES often affects the physical health of individuals through a combination of lifestyle and psychosocial factors. For example, people with lower SES are more likely to have higher rates of psychological stress and negative emotions, resulting in higher rates of smoking and drinking [54,55]. Similarly, we have noted the mediating effect of lifestyle when SES affects VI. First, nowadays, people often need to read a lot in daily life and face more social interactions that require smoking and drinking, while having less time available to exercise. However, people with higher education levels can usually better control themselves since they are generally more aware of the harms of smoking, excessive drinking and overusing eyes when reading, and the benefits of exercising. As a result, through these combined lifestyle factors, a higher level of education has a positive effect on their visual health. Second, people with higher incomes generally face fewer life pressures and do not need to dispel negative emotions through smoking or drinking. Moreover, their neighbourhood environments usually provide more infrastructure supportive of healthy lifestyles, such as parks and exercise facilities [56,57]. Thus, through these lifestyle factors, a higher income level can greatly reduce the risk of VI.

The finding that SES is significantly associated with VI is not new. However, to our knowledge, this is the first study to explore the mediating role of lifestyles between SES and VI, with certain policy implications. As opposed to previous studies that only attend to a particular age group such as the elderly or children, we have used all age groups in order to reach more general conclusions. To ensure the robustness of our results, we have also undertaken an additional subgroup analysis restricted to participants aged 16 years and above. Moreover, unlike some epidemiologic literatures on the visual health of adults, which principally identified whether participants have VI (i.e., low vision and blindness), we broke VI down to four levels based on the severity of VI and undertook more in-depth analysis through ordinal logistic regression analysis. Finally, this study used samples from developed rural areas of China, in response to a lack of relevant studies in China.

Although this study includes a large, representative and population-based design, it nonetheless has some notable limitations. First, we derived the results of our study using the cross-sectional data in a survey from November 2015 to March 2017, which may not entirely reflect other years. Second, due to the cross-sectional nature of this study, causal inferences cannot be made with complete certainty. That is to say, we may not be able to determine whether people with low SES levels are more likely to develop VI, or whether people with VI are more likely at lower SES level limited by physical condition. Third, the findings from the participants of developed rural areas of China might not be generalisable to all rural areas in China, due to differences in local healthcare systems, prevalence of eye diseases and socio-economic development in surrounding areas. Therefore, a large-scale study involving the populations of all rural areas in China is required to confirm our conclusions from developed rural areas in China. Fourth, the rationality of the questionnaire design and the reliability of the information collected by questionnaires can be questioned, and the results of this investigation may not be comparable with those of prior studies. Finally, the results of the association between SES and VI, as well as the mediating effect of different lifestyles, may differ among people in different age groups. Therefore, it is necessary to conduct more detailed studies with different age groups in the future.

## Conclusions

In conclusion, visual health was found to differ significantly among population groups with varied demographic characteristics, SES and medical histories in developed rural areas of China. VI is influenced by age, sex, the presence of some chronic diseases (include hypertension, diabetes, CT and tumour) and SES factors (i.e. education level and income level). Lifestyle factors including smoking, drinking, reading and exercising play mediating roles between SES and VI.

In light of the results from this study, some targeted public health interventions with the aim of reducing VI should be developed. First, special health services for the early diagnosis, treatment and improved follow-up of eyesight disorders among lower education and lower income populations should be provided. Second, actions to increase the awareness of people regarding the advantages and disadvantages of different lifestyles for visual health should be taken. Finally, in developed rural areas of China, further treatment of the major eye diseases that cause VI and some eye-specific strategies should be undertaken among people. For example, targeted approaches to improve early detection, such as formal screening programmes, could be put into practice. Specifically, metrics of SES factors could be included, and consequently better results could be realised within treatment and visual rehabilitation protocols.

## Supporting information

S1 Checklist STROBE checklist. (DOCX)

S2 Checklist PLOS ONE clinical studies checklist. (DOCX)

S1 Table Raw data. (XLSX)

S2 Table Income data. (XLSX)

S1 Appendix Questionnaire in original language. (DOCX)

S2 Appendix Questionnaire in English. (DOCX)
