# Supplementary material for: Socio-economic status, visual impairment and the mediating role of lifestyles in developed rural areas of China
Source: PLoS One. 2019 Apr 11;14(4):e0215329. doi: 10.1371/journal.pone.0215329 (PMC6459527; doi:10.1371/journal.pone.0215329)
Supplement: S1 Checklist — (DOCX) [file pone.0215329.s001.docx]

STROBE Statement—checklist of items that should be included in reports of observational studies

|  | Item No. | Recommendation | Page  No. | Relevant text from manuscript |
| --- | --- | --- | --- | --- |
| **Title and abstract** | 1 | (*a*) Indicate the study’s design with a commonly used term in the title or the abstract | 2 | Cross-sectional study |
|  |  | (*b*) Provide in the abstract an informative and balanced summary of what was done and what was found | 2-3 | Purpose, methods, results, conclusion |
| Introduction | | | |  |
| Background/rationale | 2 | Explain the scientific background and rationale for the investigation being reported | 4 | Visual impairment (VI) constitutes a major public health problem. The prevalence of VI has been found to be strongly associated with socio-economic status (SES). Lifestyle has been identified as one of the intermediate mechanisms that links SES with health, that is, differences in SES lead to inequalities in health through lifestyle. However, relatively little research has examined the mediating role of lifestyle factors between SES and VI. |
| Objectives | 3 | State specific objectives, including any prespecified hypotheses | 5 | To examine the effects of SES on VI using samples from developed rural areas of China, and to further analyse the mediating effects of several lifestyle factors on this relationship. Several public health interventions for reducing VI were proposed in the end. |
| Methods | | | |  |
| Study design | 4 | Present key elements of study design early in the paper | 5 | To examine the effects of SES on VI using samples from developed rural areas of China, and to further analyse the mediating effects of several lifestyle factors on this relationship. |
| Setting | 5 | Describe the setting, locations, and relevant dates, including periods of recruitment, exposure, follow-up, and data collection | 5-7 | This cross-sectional study was conducted from November 2015 to March 2017 on a Chinese population living in Tianjin, a developed municipality in Northern China. All the participants of this survey were recruited in October 2015. We had access to information that could identify individual participants after data collection. |
| Participants | 6 | (*a*) *Cohort study*—Give the eligibility criteria, and the sources and methods of selection of participants. Describe methods of follow-up  *Case-control study*—Give the eligibility criteria, and the sources and methods of case ascertainment and control selection. Give the rationale for the choice of cases and controls  *Cross-sectional study*—Give the eligibility criteria, and the sources and methods of selection of participants | 6 | *Cross-sectional study*—We conducted multistage cluster sampling with persons of all age groups in the selected districts. |
|  |  | (*b*) *Cohort study*—For matched studies, give matching criteria and number of exposed and unexposed  *Case-control study*—For matched studies, give matching criteria and the number of controls per case | 6 |  |
| Variables | 7 | Clearly define all outcomes, exposures, predictors, potential confounders, and effect modifiers. Give diagnostic criteria, if applicable | 7-10 | Refer to the section of Variables |
| Data sources/ measurement | 8* | For each variable of interest, give sources of data and details of methods of assessment (measurement). Describe comparability of assessment methods if there is more than one group | 7-10 | Refer to the section of Variables |
| Bias | 9 | Describe any efforts to address potential sources of bias | 28-29 |  |
| Study size | 10 | Explain how the study size was arrived at | 6 | Based on the formula of simple random sampling, we calculated the sample size for each age group, with a 95% confidence interval (CI), an allowable error bound of 20% and a 10% non-response rate. |

Continued on next page

| Quantitative variables | 11 | Explain how quantitative variables were handled in the analyses. If applicable, describe which groupings were chosen and why | 7-10 |  |
| --- | --- | --- | --- | --- |
| Statistical methods | 12 | (*a*) Describe all statistical methods, including those used to control for confounding | 10-11 | Descriptive analysis, χ2 test, stepwise regression method. |
|  |  | (*b*) Describe any methods used to examine subgroups and interactions | 11 | Given that most children and adolescents may continue to receive education and have no income, we undertook an additional subgroup analysis restricted to those participants who were 16 years old and above. |
|  |  | (*c*) Explain how missing data were addressed | 11 | Of a total of 15,368 participants in the four districts, 12,233 were included for final analysis having excluded those with unknown outcomes and incomplete questionnaires. |
|  |  | (*d*) *Cohort study*—If applicable, explain how loss to follow-up was addressed  *Case-control study*—If applicable, explain how matching of cases and controls was addressed  *Cross-sectional study*—If applicable, describe analytical methods taking account of sampling strategy | 6 | *Cross-sectional study* |
|  |  | (*e*) Describe any sensitivity analyses |  |  |
| Results | | | | |
| Participants | 13* | (a) Report numbers of individuals at each stage of study—eg numbers potentially eligible, examined for eligibility, confirmed eligible, included in the study, completing follow-up, and analysed | 11 | Of a total of 15,368 participants in the four districts, 12,233 were included for final analysis having excluded those with unknown outcomes and incomplete questionnaires. |
|  |  | (b) Give reasons for non-participation at each stage |  |  |
|  |  | (c) Consider use of a flow diagram |  |  |
| Descriptive data | 14* | (a) Give characteristics of study participants (eg demographic, clinical, social) and information on exposures and potential confounders | 12-14 | Distribution of demographic, socio-economic status, medical history and lifestyle variables among all participants and people with or without visual impairment. |
|  |  | (b) Indicate number of participants with missing data for each variable of interest |  |  |
|  |  | (c) *Cohort study*—Summarise follow-up time (eg, average and total amount) |  |  |
| Outcome data | 15* | *Cohort study*—Report numbers of outcome events or summary measures over time |  |  |
|  |  | *Case-control study—*Report numbers in each exposure category, or summary measures of exposure |  |  |
|  |  | *Cross-sectional study—*Report numbers of outcome events or summary measures | 14-25 | *Cross-sectional study* |
| Main results | 16 | (*a*) Give unadjusted estimates and, if applicable, confounder-adjusted estimates and their precision (eg, 95% confidence interval). Make clear which confounders were adjusted for and why they were included | 14-25 |  |
|  |  | (*b*) Report category boundaries when continuous variables were categorized | 14-25 |  |
|  |  | (*c*) If relevant, consider translating estimates of relative risk into absolute risk for a meaningful time period | 14-25 |  |

Continued on next page

| Other analyses | 17 | Report other analyses done—eg analyses of subgroups and interactions, and sensitivity analyses | 14-25 | Analyses of subgroups |
| --- | --- | --- | --- | --- |
| Discussion | | | | |
| Key results | 18 | Summarise key results with reference to study objectives | 25 | VI is associated with several key demographic (including age and sex), SES (including educational level and income level) and medical history factors, with a trend across the severity of VI. Moreover, several lifestyles were demonstrated to have mediating effects between SES and VI. |
| Limitations | 19 | Discuss limitations of the study, taking into account sources of potential bias or imprecision. Discuss both direction and magnitude of any potential bias | 28-29 | Although this study includes a large, representative and population-based design, it nonetheless has some notable limitations. |
| Interpretation | 20 | Give a cautious overall interpretation of results considering objectives, limitations, multiplicity of analyses, results from similar studies, and other relevant evidence | 25-28 |  |
| Generalisability | 21 | Discuss the generalisability (external validity) of the study results | 28 |  |
| Other information | |  | | |
| Funding | 22 | Give the source of funding and the role of the funders for the present study and, if applicable, for the original study on which the present article is based |  | In the Financial Disclosure. |

*Give information separately for cases and controls in case-control studies and, if applicable, for exposed and unexposed groups in cohort and cross-sectional studies.

**Note:** An Explanation and Elaboration article discusses each checklist item and gives methodological background and published examples of transparent reporting. The STROBE checklist is best used in conjunction with this article (freely available on the Web sites of PLoS Medicine at http://www.plosmedicine.org/, Annals of Internal Medicine at http://www.annals.org/, and Epidemiology at http://www.epidem.com/). Information on the STROBE Initiative is available at www.strobe-statement.org.
