# Supplementary material for: Socio-economic status, visual impairment and the mediating role of lifestyles in developed rural areas of China
Source: PLoS One. 2019 Apr 11;14(4):e0215329. doi: 10.1371/journal.pone.0215329 (PMC6459527; doi:10.1371/journal.pone.0215329)
Supplement: S1 Appendix — (DOCX) [file pone.0215329.s005.docx]

**天津市视力健康状况问卷与调查表**

编号

**一、基本情况**（在与您相符的项目上划 “√”）

姓名 年 龄 出生年月 性别（1）男（2）女

联系方式 家庭住址

1.婚姻状况 （1）未婚 （2）已婚 （3）未婚同居 （4）离婚 （5）丧偶

2.文化程度 （1）文盲 （2）小学 （3）初中 （4）高中或中专

 （5）大学或大专、研究生及以上

3.民 族 （1）汉 （2）其他

4.职 业 （1）专业技术人员 （2）机关干部 （3）办事人员 （4）工人 （5）军人

 （6）商业人员 （7）服务人员 （8）农林牧渔 （9）其它 （10）失业

5.家庭每月人均收入

（1）无收入（2）1000元以下（3）1000-2000元（4）2000-5000元（5）5000元以上

**二、生活方式及病史**（在与您相符的项目上划 “√”）

6.您是否有以下行为？

（1）阅读时间

①从不 ②每周 ③1小时以下/天 ④1-2小时/天 ⑤2小时以上/天

（2）看显示屏时间 （包括电脑、电视、游戏机、手机等）

①从不 ②每周 ③1小时以下/天 ④1-2小时/天 ⑤2小时以上/天

（3）运动时间

①从不 ②每周 ③1小时以下/天 ④1-2小时/天 ⑤2小时以上/天

7.您的工作和生活环境是否接触高强度光照及紫外线等？

（1）否 （2）是

8.您是否有如下的嗜好和生活习惯？

（1）抽烟 （2）喝酒 （3）偏食 （4）熬夜、睡眠不足 （5）其他

9.您是否被确诊患有如下疾病？

（1）高血压 ①无 ②有 年 血压 mmHg

（2）糖尿病 ①无 ②有 年 血糖 mmol/L

（3）心脏病 ①无 ②有 年 （4）脑梗塞 ①无 ②有 年

（5）脑外伤 ①无 ②有 年 （6）手术史 ①无 ②有 年

（7）肿瘤 ①无 ②有 年 （8）其他 ①无 ②有 年

10.您是否被确诊患有如下眼病？

（1）近视/远视/散光 （2）斜视与弱视 （3）白内障 （4）糖尿病视网膜病变

（5）黄斑变性 （6）色素膜炎 （7）青光眼 （8）角膜炎/角膜混浊

（9）视神经病变 （10）视网膜脱离 （11）眼外伤 （12）视网膜色素变性

（13）眼球萎缩/无眼球（14）其它

11.家族病史及遗传病史

（1）白内障家族史 ①无 ② 有 人

（2）青光眼家族史 ①无 ② 有 人

（3）玻璃体视网膜疾病家族史 ①无    ② 有 人

（4）斜视与弱视家族史 ①无 ② 有 人

（5）其他遗传病史

**三、相关眼部检查**

1. 眼位：（1）眼位正 （2）眼位偏斜
2. 视力检查（记录PVA）

（1）≥5岁 ：裸眼视力 右： ；左： ； 最佳矫正视力 右： ；左： ；

（2）1～4岁：① HOTV字母视力表 右： ；左：

②在3米处深色背景上滚动一乒乓球，令其拾起，注意眼球是否注意滚动的乒乓球。a. 正常 b.可疑

（3）＜1岁 ：①眼球有无浮动或上翻？ a. 正常 b.可疑

②外观有无影响视力的异常？ a. 正常 b.可疑

③能否注视玩具？能否追手电光？ a. 正常 b.可疑

3．若最佳矫正视力PVA＜6/18，做裂隙灯、眼底镜检查如下：

角膜：（1）透明 （2）混浊或溃疡 （3）云翳

前房：（1）深 （2）正常 （3）浅； 房水：（1）清亮 （2）混浊

瞳孔：形态：（1）圆形 （2）欠圆； 大小：（1）大 （2）正常 （3）小；

直接对光反射：（1）灵敏（2）迟钝（3）消失；间接对光反射：（1）灵敏（2）迟钝（3）消失

晶状体：（1）透明 （2）混浊

玻璃体：（1）透明 （2）混浊

视网膜：视乳头：颜色： ；边界： ；血管管径 动脉： ；静脉： ；走形： ；

4．特殊检查：（1）散瞳查眼底 ：病变 ；

（2）眼压： 右眼： mmHg；左眼： mmHg；

（3）视野半径： 右眼 ；左眼 ；

（4）眼B超： 右眼： ；左眼： ；

5．视力损伤的分级：

（1）1级 （2）2级 （3）3级 （4）4级

6．推断引起视力残疾的主要原因：

| （1）白内障 | （2）青光眼 | （3）屈光不正/弱视 | （4）糖尿病视网膜病变 |
| --- | --- | --- | --- |
| （5）年龄相关性黄斑病变 | （6）色素膜炎 | （7）斜视与眼球震颤 | （8）角膜混浊/角膜疤痕 |
| （9）眼外伤 | （10）视网膜脱离 | （11）视神经萎缩 | （12）视网膜色素变性 |
| （13）眼球萎缩/无眼球 | （14）小眼球/小角膜 | （15）白内障术后后囊混浊 | （16）其它 |

调查员

附:视力损伤的分级标准(世界卫生组织，1973)

| **视力损伤级别** | | **较好眼最佳矫正视力（PVA）** |
| --- | --- | --- |
| **类别** | **级别** |  |
| 低视力 | 1级（中度视力残疾） | 6/60≤PVA<6/18 |
|  | 2级（严重视力残疾） | 3/60≤PVA<6/60 |
| 盲 | 3级（重度视力残疾） | 12/600≤PVA<3/60 |
|  | 4级（接近完全失明） | PVA<12/600 |
