# Supplementary material for: Socio-economic status, visual impairment and the mediating role of lifestyles in developed rural areas of China
Source: PLoS One. 2019 Apr 11;14(4):e0215329. doi: 10.1371/journal.pone.0215329 (PMC6459527; doi:10.1371/journal.pone.0215329)
Supplement: S2 Appendix — (DOCX) [file pone.0215329.s006.docx]

**Questionnaire of Vision Health Status in Tianjin**

**No.**

**1. General Information** (Tick the options that match yours)

Name Age Date of Birth Sex ① Male ② Female

Mobile Number Address

(1) Marital status ① Single ② Married ③ Cohabit ④ Divorced ⑤ Widowed

(2) Education level ① No studies ② Primary ③ Junior ④ Senior ⑤ University and above

(3) Race ① Han ② Others

(4) Occupation ① Professional ② Government functionary ③ Clerk ④ Worker

⑤ Soldier ⑥ Commercial staff ⑦ Service staff ⑧ Staff of agriculture, forestry, animal husbandry and fishery ⑨ Others ⑩ Unemployed

(5) Per capita household income per month ① No income ② Up to 1,000 yuan

③ 1,000-2,000 yuan ④ 2,000-5,000 yuan ⑤ More than 5,000 yuan

**2. Lifestyles and Medical Histories** (Tick the options that match yours)

(6) Do you have the following behaviors?

- Frequency of reading

1. Never ② Every Week ③ Less than 1h/day ④ 1-2h/day ⑤ More than 2h/day

- Frequency of looking at screen (such as computer, TV, PSP, mobile phone, etc.)

1. Never ② Every Week ③ Less than 1h/day ④ 1-2h/day More than 2h/day

- Frequency of doing physical exercise

① Never ② Every Week ③ Less than 1h/day ④ 1-2h/day ⑤ More than 2h/day

(7) Do you work or live in an environment that exposed to high intensity light, ultraviolet light, etc.?

① No ② Yes

(8) Do you have the following living habits？

① Smoking ② Drinking ③ Picky eater ④ Staying up late ⑤ Others

(9) Have you been diagnosed with the following diseases?

| Hypertension ① No ② Yes years | Blood Pressure mmHg |
| --- | --- |
| Diabetes ① No ② Yes years | Blood Glucose mmol/L |
| Cardiopathy ① No ② Yes years | Cerebral Infarction ① No ② Yes years |
| Cerebral Trauma ① No ② Yes years | Operations ① No ② Yes years |
| Tumour ① No ② Yes years | Others ① No ② Yes years |

(10) Have you been diagnosed with the following eye diseases?

① Myopia/Hyperopia/Astigmatism ② Strabismus and Refractive Error ③ Cataract

④ Diabetic Retinopathy ⑤ Age-Related Macular Degeneration ⑥ Uveitis

⑦ Glaucoma ⑧ Nubecula ⑨ Optic Atrophy ⑩ Amotio Retinae

Ocular Traumas Retinitis Pigmentosa Atrophy of Eyeball Others

(11) Family medical histories and genetic histories

- Family history of Cataract ① No ② Yes people
- Family history of Glaucoma ① No ② Yes people
- Family history of Vitreoretinal Disease ① No ② Yes people
- Family history of Strabismus and Refractive Error ① No ② Yes people
- Other genetic histories

**3. Examination of Eyes** (Tick the options that match yours)

(1) Eye Position: ① Normal eye position ② Deviation of eye position

(2) Examination of visual acuity (Presenting Visual Acuity (PVA) recorded)

- ≥5 years old:

① Uncorrected PVA Right Left ② Best Corrected PVA Right Left

- 1～4 years old: ① HOTV Cards Right Left

② Roll a table tennis ball on a dark background 3 meters away. Ask him (her) to pick up the table tennis and pay attention to whether the eyeballs pay attention to the rolling table tennis. a. Normal b. Questionable

- ＜1 years old: ① Does the eyeball float or roll up? a. Normal b. Questionable

② Does the appearance have the abnormality that affects visual acuity? a. Normal b. Questionable ③ Can he (she) stare at the toy? Can he (she) track the flashlight? a. Normal b. Questionable

(3) For the participants with PVA＜6/18 in either eye, perform slit-lamp biomicroscopy and direct ophthalmoscopy as follows:

- Cornea: ① Transparent ② Corneal Opacity or Ulcer ③ Nebula
- Anterior Chamber: ① Deep ② Normal ③ Shallow
- Aqueous Humor: ① Clear ② Opacity
- Pupil: Shape: ① Round ② Out-of-round Size: ① Big ② Normal ③ Small
- Direct light pupillary reflex: ① Sensitive ② Insensitive ③ Disappear
- Indirect light pupillary reflex: ① Sensitive ② Insensitive ③ Disappear
- Lens: ① Transparent ② Opacity
- Vitreous Body: ① Transparent ② Opacity
- Retina: Optic nerve head: Color Boundary ; Vascular Caliber: Artery Vein ; Deformation:

(4) Special Examination:

| ① Dilated for Fundus Examination: Lesion | |
| --- | --- |
| ② Intraocular Pressure: Right mmHg; Left mmHg | |
| ③ Field of Vision Radius: Right ; Left | ④ B ultrasound of Eye: Right ; Left |

(5) Level of Visual Impairment: ① 1^st^ ② 2^nd^ ③ 3^rd^ ④ 4^th^

(6) Inference of the main causes of visual impairment

| ① Cataract | ② Glaucoma | ③ Refractive Error | ④ Diabetic Retinopathy | |
| --- | --- | --- | --- | --- |
| ⑤ Age-Related Macular Degeneration | | ⑥ Uveitis | ⑦Strabismus and Nystagmus | |
| ⑧ Nubecula | ⑨ Ocular Traumas | ⑩ Amotio Retinae | Optic Atrophy | |
| Retinitis Pigmentosa | | Atrophy of Eyeball | | |
| Microphthalmus/ Microcornea | | Posterior Capsule Opacification | | Others |

**Recorder**

***Attachment: Grading Criteria for Visual Impairment*** (WHO, 1973)

| **Levels of Visual Impairment** | | **Best Corrected Presenting Visual Acuity (PVA) in the Better eye** |
| --- | --- | --- |
| **Categories** | **Levels** |  |
| Low Vision | 1^st^ (Moderate Visual Impairment) | 6/60≤PVA<6/18 |
|  | 2^nd^ (Severe Visual Impairment) | 3/60≤PVA<6/60 |
| Blindness | 3^rd^ (Profound Visual Impairment) | 12/600≤PVA<3/60 |
|  | 4^th^ (Near Total Blindness) | PVA<12/600 |
